# Ergosta-7,9(11),22-trien-3β-ol Rescues AD Deficits by Modulating Microglia Activation but Not Oxidative Stress

**DOI:** 10.3390/molecules26175338

**Published:** 2021-09-02

**Authors:** Hsin-Ping Liu, Yueh-Hsiung Kuo, Jack Cheng, Li-Zhong Chang, Meng-Shiun Chang, Li-Wen Su, Tsai-Ni Chuang, Wei-Yong Lin

**Affiliations:** 1 Graduate Institute of Acupuncture Science, College of Chinese Medicine, China Medical University, Taichung 40402, Taiwan; hpliu@mail.cmu.edu.tw (H.-P.L.); bluefish5341@gmail.com (L.-W.S.); 2 Department of Chinese Pharmaceutical Sciences and Chinese Medicine Resources, College of Chinese Medicine, China Medical University, Taichung 40402, Taiwan; kuoyh@mail.cmu.edu.tw; 3 Department of Biotechnology, Asia University, Taichung 41354, Taiwan; 4 Chinese Medicine Research Center, China Medical University, Taichung 404, Taiwan; 5 Graduate Institute of Integrated Medicine, College of Chinese Medicine, China Medical University, Taichung 40402, Taiwan; t91917@mail.cmuh.org.tw (J.C.); mq135280@hotmail.com (L.-Z.C.); cherry_accio@hotmail.com (M.-S.C.); e96541230@gmail.com (T.-N.C.); 6 Department of Medical Research, China Medical University Hospital, Taichung 40447, Taiwan; 7 Brain Diseases Research Center, China Medical University, Taichung 40402, Taiwan

**Keywords:** Alzheimer’s disease, *Drosophila*, EK100, ergosta-7,9(11),22-trien-3β-ol

## Abstract

Ergosta-7,9(11),22-trien-3β-ol (EK100) was isolated from the Taiwan-specific medicinal fungus Antrodia camphorata, which is known for its health-promotion and anti-aging effects in folk medicine. Alzheimer’s disease (AD) is a major aging-associated disease. We investigated the efficacy and potential mechanism of ergosta-7,9(11),22-trien-3β-ol for AD symptoms. *Drosophila* with the pan-neuronal overexpression of human amyloid-β (Aβ) was used as the AD model. We compared the life span, motor function, learning, memory, oxidative stress, and biomarkers of microglia activation and inflammation of the ergosta-7,9(11),22-trien-3β-ol-treated group to those of the untreated control. Ergosta-7,9(11),22-trien-3β-ol treatment effectively improved the life span, motor function, learning, and memory of the AD model compared to the untreated control. Biomarkers of microglia activation and inflammation were reduced, while the ubiquitous lipid peroxidation, catalase activity, and superoxide dismutase activity remained unchanged. In conclusion, ergosta-7,9(11),22-trien-3β-ol rescues AD deficits by modulating microglia activation but not oxidative stress.

## 1. Introduction

Antrodia camphorata is a medicinal fungus unique to Taiwan, and was an important economic crop for Taiwan’s foreign trade in the 19th century [1]. Taiwan’s first history book, Tâi-uân Thong-sú (literally the General History of Taiwan), recorded the indigenous medical uses of Antrodia camphorata, including eliminating pathogens, “improving health”, “invigorating the blood”, “alcohol detoxification”, “protecting the liver”, and “anti-aging” [2,3,4]. Since we face a global population health challenge due to aging [5], the scientific study of indigenous medicine to harness anti-aging knowledge is crucial.

Among the major aging-associated diseases, such as macular degeneration, atherosclerosis, cancer, and stroke, Alzheimer’s disease (AD) is a hard nut to crack due to its elusive pathological mechanism. Effective disease-modifying treatment for AD is still unavailable [6]. Current pathological hypotheses of AD include, but are not restricted to, reductions in choline acetyltransferase, glutamate excitotoxicity, amyloid beta (Aβ) aggregation, tau phosphorylation, brain insulin resistance [7], and gut microbiome alterations [8]. Nevertheless, no matter the kind of AD-initiating causes are involved, oxidative stress and inflammatory microglia activation are the common signs accompanying the neurodegeneration of AD [9,10]. Interestingly, neuroinflammation and oxidative stress are interrelated events which are both observed in AD and hypothesized in AD pathology. On the one hand, senile plaques of Aβ deposition trigger inflammatory reactions, microglia activation, cytokine release, and astrocytosis, leading to undesirable consequences such as progressive neuronal injury and, finally, cognitive dysfunctions. The activation of immune responses may initially combat invading pathogens with Aβ or eliminate excessive Aβ deposits; however, the process inevitably produces reactive oxygen species (ROS), which may cause neuronal damage in the microenvironment. On the other hand, Aβ could cause ROS in an inflammation-independent manner. For example, Aβ was found in the mitochondrial membranes of AD postmortem and could enter the mitochondria, causing the disruption of the electron transport chain (ETC) and resulting in ROS generation. Elevated ROS may, in turn, trigger cytokine release and initiate inflammation [7,9,10]. Therefore, anti-oxidative and ant-inflammatory molecules might be potential therapeutics for AD.

Antrodia camphorate, especially one of its submerged whole-broth-isolated compounds ergosta-7,9(11),22-trien-3β-ol (also known as EK100), exhibited profound anti-oxidative and anti-inflammatory activities [11,12,13]. Furthermore, Chang et al. [14] have shown that antroquinonol, another compound isolated from the fermented mycelium of Antrodia camphorate, improves learning and memory in an AD mouse model with lowered astrogliosis and oxidative stress. However, the causal relationships between the reduced astrogliosis and oxidative stress upon antroquinonol treatment could not be confirmed, and the lowering of oxidative stress has been postulated as the mediating factor [14]. The safety of EK100 has been tested previously following the Organization for Economic Co-operation and Development (OECD) Guideline 407 in a mice model of 10 and 20 mg/kg-day [15]. Therefore, we adopted the approximately equivalent concentrations of free feeding for fruit flies in this study.

The fruit fly (*Drosophila melanogaster*) model of Aβ42 overexpression is a well-established AD animal model [16], with characteristic AD symptoms including reduced life span, learning and motor deficits [17], oxidative stress [18], and microglial activation [19]. Modeling AD’s challenges in *Drosophila melanogaster* include no BACE activity in *Drosophila* and gender differences, which show more severe symptoms in male files. These are not observed in human AD [20]. Therefore, we adopted human Aβ42 peptide expressing flies to model AD, where Aβ42 production requires no BACE activity. In this study, we tested whether EK100 improves AD symptoms, and more importantly, whether both modulations of oxidative stress and astrogliosis are essential to ameliorate AD.

## 2. Results

### 2.1. Ergosta-7,9(11),22-trien-3β-ol Rescued Survival and Climbing of AD Drosophila

**Figure 1:**
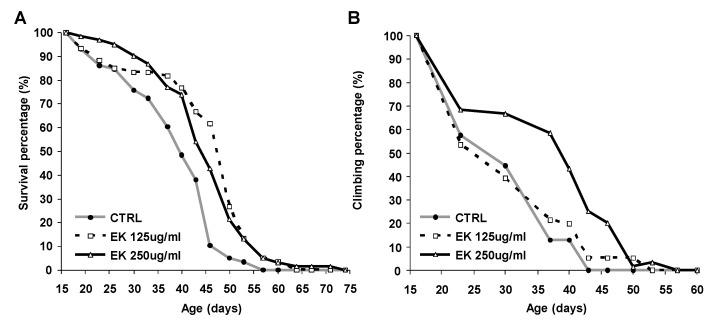
Ergosta-7,9(11),22-trien-3β-ol improved the survival and climbing of AD Drosophila. (**A**) Survival curve of AD Drosophila treated with different doses of ergosta-7,9(11),22-trien-3β-ol. (**B**) Climbing curve of AD Drosophila treated with different doses of ergosta-7,9(11),22-trien-3β-ol. CTRL denotes drug-free control, while EK denotes ergosta-7,9(11),22-trien-3β-ol (EK100).

AD diagnosis is associated with a reduction in median life span [21], and nearly half of AD patients suffer from activity disturbances [22]. Therefore, we tested whether the Antrodia camphorata extract ergosta-7,9(11),22-trien-3β-ol (EK100) improves the survival and climbing ability of an AD Drosophila model. We found that treatment with 125 or 250 μg/mL of ergosta-7,9(11),22-trien-3β-ol improved the survival of AD Drosophila significantly (*p* < 0.01) (Figure 1A and Supplementary Table S1), but that only a high dose (250 μg/mL) improved the climbing of AD Drosophila (Figure 1B). Since survival and mobility deficits are clinical symptoms of AD, the 250 μg/mL concentration, which improved both symptoms, was used in the following investigations.

### 2.2. Ergosta-7,9(11),22-trien-3β-ol Rescued Learning and One-Hour Memory of AD Drosophila

**Figure 2:**
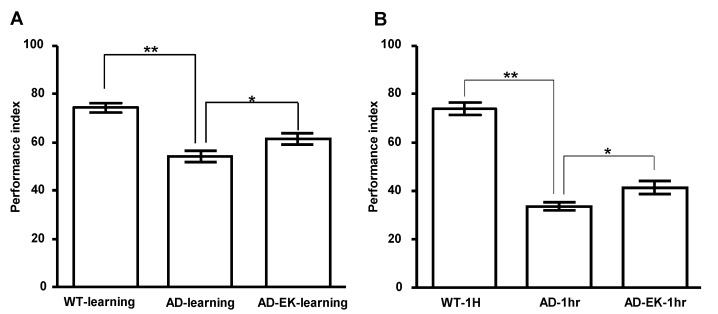
Ergosta-7,9(11),22-trien-3β-ol improved (**A**) the learning ability and (**B**) one-hour memory of AD Drosophila. Error bar stands for standard error of mean, while * or ** denote *p* < 0.05 or 0.01 in Student’s *t*-test, respectively. WT denotes wildtype control. AD denotes Alzheimer’s model. AD-EK denotes the Alzheimer’s model treated with ergosta-7,9(11),22-trien-3β-ol (EK100).

Next, we tested whether ergosta-7,9(11),22-trien-3β-ol treatment improves learning and memory defects, a hallmark symptom of AD. AD flies were treated with ergosta-7,9(11),22-trien-3β-ol at 250 μg/mL for 14 days and subjected to learning and memory tests with T-maze. As shown in Figure 2A, compared to the wildtype control (AD(-)/EK(-)), the learning ability of the AD control (AD(+)/EK(−)) declined significantly (*p* < 0.01), while ergosta-7,9(11),22-trien-3β-ol treatment significantly improved the learning ability of AD (*p* < 0.05). Moreover, ergosta-7,9(11),22-trien-3β-ol treatment also improved the declined one-hour memory of AD (Figure 2B).

### 2.3. Ergosta-7,9(11),22-trien-3β-ol Modulated Microglial Activation in AD Drosophila

**Figure 3:**
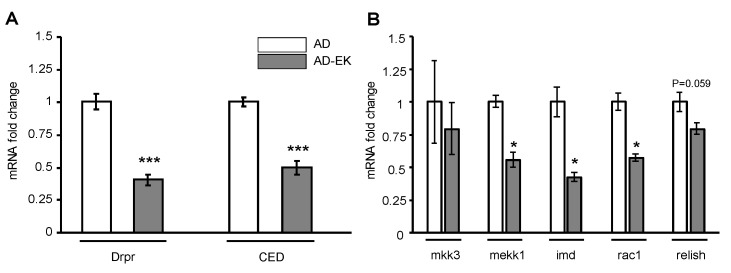
Ergosta-7,9(11),22-trien-3β-ol decreased the expression of (**A**) microglial activation and (**B**) inflammatory markers. Error bar stands for standard error of mean, while * and *** denotes *p* < 0.05 and 0.001 in Student’s *t*-test, respectively. AD denotes Alzheimer’s model. AD-EK denotes Alzheimer’s model treated with ergosta-7,9(11),22-trien-3β-ol (EK100).

Next, we tested whether ergosta-7,9(11),22-trien-3β-ol treatment decreases microglial activation in AD. In AD, activated microglia are accompanied by phagocytosis and neurodegeneration [23]. In Drosophila, the microglia cell engulfment marker, and hence the microglial activation marker, is the expression of draper (Drpr) and the downstream CED [24]. After ergosta-7,9(11),22-trien-3β-ol treatment, AD flies were culled, and RNA was extracted from the head of flies for RT-qPCR. As shown in Figure 3A, both Drpr and CED were significantly down-regulated in ergosta-7,9(11),22-trien-3β-ol-treated AD Drosophila compared to the untreated AD. This implies that ergosta-7,9(11),22-trien-3β-ol decreased microglial activation in AD Drosophila. Moreover, ergosta-7,9(11),22-trien-3β-ol treatment also lowered the CNS inflammation, as revealed in the down-regulation of genes related to inflammation (Figure 3B).

### 2.4. Ergosta-7,9(11),22-trien-3β-ol Did Not Modulate Ubiquitous Oxidative Stress in AD Drosophila

**Figure 4:**
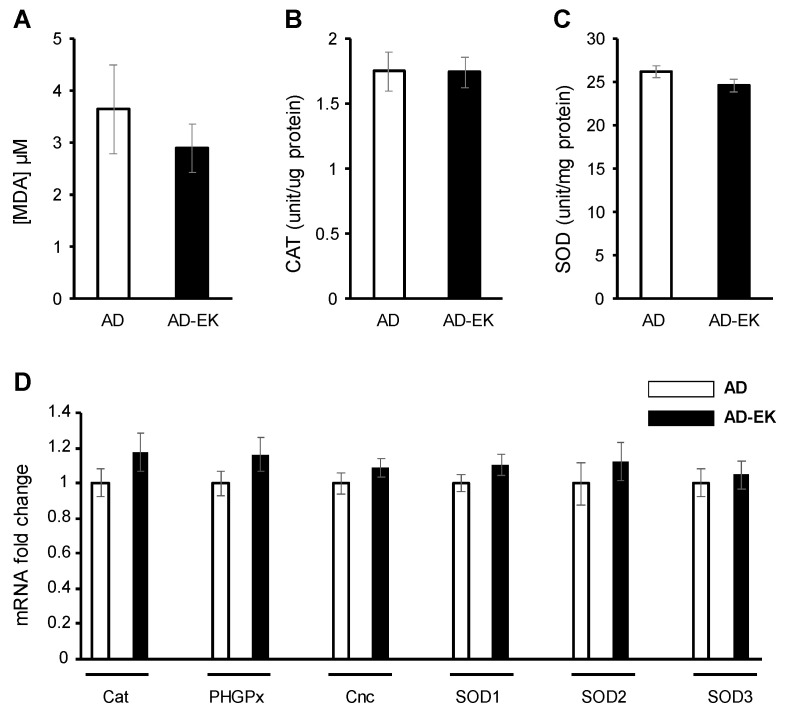
Ergosta-7,9(11),22-trien-3β-ol did not modulate oxidative stress in an AD fruit fly model. (**A**) Lipid peroxidation (MDA) assay. (**B**) Catalase assay. (**C**) SOD assay. (**D**) mRNA expression of oxidative stress genes. Error bar stands for standard error of mean. AD denotes Alzheimer’s model. AD-EK denotes Alzheimer’s model treated with ergosta-7,9(11),22-trien-3β-ol (EK100).

Next, we tested whether the modulation of ubiquitous—i.e., whole body—oxidative stress is necessary for ergosta-7,9(11),22-trien-3β-ol to rescue AD. Ergosta-7,9(11),22-trien-3β-ol treatment did not alter the lipid peroxidation (Figure 4A), catalase activity (Figure 4B), superoxide dismutase activity (Figure 4C), or the expression of oxidative stress-related genes (Figure 4D).

## 3. Discussion

This study reports the efficacy and possible underlying mechanism of ergosta-7,9(11),22-trien-3β-ol, an extract from *Antrodia camphorata*, for the symptom modification of AD using the Drosophila model with human-Aβ overexpression. Ergosta-7,9(11),22-trien-3β-ol effectively improved learning and memory and extended the life span of patients with AD. In addition, biomarkers of microglia activation and inflammation were reduced, while ubiquitous oxidative stress was unchanged.

There are many sources of oxidative stress in AD, including but not limited to the activation of innate immunity—for example, Aβ accumulation, the disruption of mitochondria by Aβ, hyperphosphorylated tau, and metal malmetabolism are also sources [25]. Although inflammation and oxidative stress may reciprocally induce each other, we may assume that their synchronous changes only occur under circumstances in which other sources of oxidative stress stay constant. However, this is obviously not the case in this study. A decrease in the innate immunity, especially microglia activation, at the same time attenuates its contribution to the clearance of Aβ, which may result in Aβ accumulation. Intriguingly, Aβ accumulation itself triggers the production of oxidative stress in multiple ways. Thus, EK100′s decreasing microglial activation and inflammatory responses may in part protect neurons from off-target immune attack but will pay the price of the attenuation of Aβ clearance. Thus, this study’s outcome may reflect the limitation of the microglia-targeting strategy, at least in the AD Drosophila model.

The microglia-mediated innate immune system is described as a double-edged sword in neurodegenerative diseases, especially AD [26,27]. Activated microglia tries to remove toxic pathogens such as Aβ, but inevitably causes neuronal damage in the microenvironment. The superiority of 250 μg/mL EK100 within 35 days may imply that the strategy of lowering microglia activation is pathological-stage-limited, at least in the case of the fruit fly AD model. Moreover, the significant lowering of biomarkers for microglial activation and inflammatory response have failed to translate into a similarly significant improvement in survival, and CNS function could be partly explained by the double-edged-sword feature of microglia. This implies that microglia activation is neither the only nor dominating factor of AD progress. Lowered microglial activation also implies that the degradation of the overexpressed Aβ was partially turned off in the fruit fly model, which may also explain the higher mortality for the higher EK100 concentration between the ages of 35 and 52 days.

Antroquinonol, another extract from Antrodia camphorata, has been reported for its efficacy on AD mice treatment, for which the lowering of oxidative stress has been postulated as the mediating factor [14]. Therefore, this may imply that antroquinonol and ergosta-7,9(11),22-trien-3β-ol improve AD symptoms through independent pathways, mediating oxidative stress or not, respectively. Hence, it would be interesting to test whether co-treatment with antroquinonol and ergosta-7,9(11),22-trien-3β-ol would have a synergetic effect on AD improvement.

For the early stage of AD, the main focus of drug design is eliminating Aβ deposition, (such as in the recently approved monoclonal antibody aducanumab [28], despite its controversy of minimal clinical benefits and ineffectiveness in one of the two phase III clinical trials [29]), or attempts to increase microglial uptake of Aβ [30,31,32]. However, for moderate or advanced stages of AD, the over-activation of microglia conversely aggravates neurodegeneration [33], and therefore alternative therapeutic strategies are required.

## 4. Materials and Methods

### 4.1. Fly Stock Maintenance and Lifespan Measurement

The Aβ transgenic Drosophila strain H29.3/CyO was obtained from [16] and was outcrossed with w(CS10) for six generations. The pan-neural driver elav-GAL4c155 line was outcrossed with w(CS10) for six generations. The white eye w1118 line (Bloomington Drosophila Stock Center #3605) outcrossed with Canton S line (Bloomington Drosophila Stock Center # 64349) 10 times (w (CS10)) was used as the standard stock. The pan-neural driver elav-GAL4c155 line was obtained from the Bloomington Drosophila Stock Center (#458). The genotype of the experimental flies—i.e., the AD model—was [elav;H29.3/+], which was selected from the F1 offspring without the CyO marker. The flies were maintained in cornmeal standard media at 25 °C under a 12 h light–dark cycle. For T-maze, the emergent [elav;H29.3/+] flies were collected (both sexes mixed and not virgin) and cultured with or without EK100 treatment until d14 after emergence. For survival, climbing, and RT-qPCR, only the male flies were used. RNA samples were collected at d30 after emergence, when both the life span and climbing curves showed significant separation compared to the untreated AD model.

In a lifespan analysis, twenty to thirty flies were raised in a food vial with or without CS; at least four vials were prepared for each treatment. Food vials were replaced every 2 to 3 days, and dead flies were counted at that time. At least three replicates were conducted per trial. The survival percentage is defined as the number of flies alive divided by the total number of flies at the beginning of the test.

### 4.2. Ergosta-7,9(11),22-trien-3β-ol (Ek100)

Ergosta-7,9(11),22-trien-3β-ol was extracted from the kind gift from Antrodia camphorata, as described previously [34]. Ergosta-7,9(11),22-trien-3β-ol forms a powder of light yellow color at room temperature. The stock solution was prepared at a concentration of 6.25 mg/mL in EtOH right before use. For lifespan and antigeotaxis assays, the final concentrations were 125 and 250 μg/mL. For learning memory and gene expression assays, the final concentration was 250 μg/mL. The molecular weight of EK100 is 396.65. So the corresponding molar concentrations are 315 or 630 μM, respectively. Flies were treated with two doses of EK100 with the final concentrations of 125 and 250 μg/mL mixed in the food by Ad libitum feeding. The control food contained the same amount of solvent EtOH as the experiment groups with the final EtOH concentration of 3% *v/v*. The treatment started from the emergence until the data collection time point of each assay. For survival and climbing assay, the treatment was given throughout the life span. The number of flies for each replication was described in the method section of each assay. We also tested lifespan assays with treated/untreated wild-type flies to ensure the safety of the ergosta-7,9(11),22-trien-3β-ol in fly models (Supplementary Figure S1).

### 4.3. Antigeotaxis Assays

The present protocol is based on a previously published method [35], with some modifications. Briefly, a transparent cylindrical plastic vial with a diameter of 2.5 cm and a height of 9.5 cm (longer will be fine, but will be more difficult to handle) was used as the geotaxis apparatus. The bottom of the vial was coated with a single layer of fly food as a cushion. A 5 cm mark above the cushion was attached to the outer surface of the vial. The other end of the vial was open, and flies were loaded from there. Since the assay was conducted concurrently to the survival assay, the number of the loaded flies was identical to the surviving ones at that moment. The flies were loaded into the vial; the top was sealed with a cap and the bottom of the vial was repeatedly (for two seconds) knocked against the hard surface on which it was standing (laboratory bench in this case). The knocking was strong enough to startle the flies and make them climb along the vial walls, but not so strong as to physically harm them. The whole process was video-recorded for 18 secs, with the number of flies climbing above the 5 cm mark noted and expressed as a percent for each group. The number of flies that climbed over the 5 cm mark were counted. Four replications were conducted for each group. The percentage of climbing ability is defined as the number of flies climbing divided by the total number of flies at the beginning of the test. Climbing ability was also tested in treated/untreated wild-type flies to ensure the safety of ergosta-7, 9 (11), 22-trien-3β-ol in fly models (Supplementary Figure S2).

### 4.4. Drosophila Learning and Memory Platform (T-Maze)

To observe the Drosophila learning and memory, about 100 flies were first placed in the upper cupper chamber, which can be supplied with electric voltage and deliver an electric shock to flies. Flies were first delivered odor 1 (MCH) along with electric shock waves. Then flies were delivered odor 2 (OCT) without electric shock waves. After training, the flies were moved to the space below, in which two open sides were supplied with either odor 1 or odor 2, respectively. By observing the moving direction of flies, the bias of odor 2 over odor 1 can be estimated with the Performance Index (PI), defined as the difference of number of flies between two odors divided by total number of flies. The Performance Index represents the ability of learning and memory of Drosophila [36]. Another group of flies was conditioned with the reversed odor pairings such that each odor served one time as electric shock + and one time as electric shock −. The PIs of these two tests were averaged as the final PI of one replicate. “1 h-memory” was tested after 1 h spacing to the training, while “learning” was, in contrast tested after 1.5 min spacing to the training. We also tested the avoidance to MCH and OCT of the AD flies with or without EK100 treatment to ensure that the treatment does cause bias against the aversive odors (Supplementary Figure S3).

### 4.5. Quantitative PCR

Heads and bodies were separated before RNA extraction. For one replicate, the flies were anaesthetized with CO_2_; collected, 100 at a time, in Eppendorf tubes; and then immersed in liquid nitrogen for 1 min to make the bodies brittle. The resulting frozen cadavers were then loaded onto stacks of sieve shakers (Bunsekifurui, No. 40 and No. 25) and agitated. The bodies broke apart and were sorted according to the size of the sieve meshes into two separate fractions: “heads” and “bodies”. The total RNA was extracted with RNeasy Mini kit (Qiagen, Düsseldorf, Germany), and then used for synthesizing cDNA with VersoTM cDNA Synthesis Kit (Thermo Scientific, Waltham, MA, USA). The RNA expression levels of the investigated genes were quantified by real-time PCR (Applied Biosystems 7700, Thermo Scientific, Waltham, MA, USA) with the Maxima SYBR Green qPCR Master Mix (Thermo Scientific, Waltham, MA, USA). The results were normalized to the relative amount of Gapdh or Rpl32. The primers used are listed below. Gapdh: 5′-GAAAAAGCGGCAGTCGTAAT-3′ and 5′-AATTCCGATCTTCGACATGG-3′; Drpr: 5′-TGTGATCATGGTTACGGAGGAC-3′ and 5′-CAGCCGGGTGGGCAA-3′; CED: 5′-CGTTTACAAGGAGCGACT-3′ and 5′-TTCCCAGATTGAAGAGCAGG-3′; Cat: 5′-TGACTACAAAAACTCCCAAACG-3′ and 5′-TTGATTCCAATGGGTGCTC-3′; PHGPx: 5′-TGACATCGGCGAGGTGT-3′ and 5′-CGGTCTGCTTGGCCTTTA-3′; Cnc: 5′-GCCAACTATGGTGGTGGAGT-3′ and 5′-ACGCTGCGATTCAGACG-3′; SOD1: 5′-GTCGACGAGAATCGTCACCT-3′ and 5′-GGAGTCGGTGATGTTGACCT-3′; SOD2: 5′-AAATTTCGCAAACTGCAAGG-3′ and 5′-GGTCGCCATTTGTTGCTATT-3′; SOD3: 5′-TCAGCATGGGTGCTCACTAT-3′ and 5′-TAATGCCCGTGGAGTTGG-3′; mkk3: 5′-CCGCTACCCATACGACAAT-3′ and 5′-GAATTCCGGCGAAAATGT-3′; mekk1: 5′-TTTAACGGCAGTGGAACTGT-3′ and 5′-TGCATCTGCAACTGCTCAC-3′; imd: 5′-AGATCGACCAGGCCATAATC-3′ and 5′-AATCCACTGGAGCAACAGC-3′; rac1: 5′-CCGTGTTCGACAACTACTCG-3′ and 5′-AGTCGGTCGTAGTCCTCCTG-3′; relish: 5′-GCCATACTCCCTTGGAATTG-3′ and 5′-TCTCCCTTCTCCGGATACAC-3′; Rpl32: 5′-CGGATCGATATGCTAAGCTGT-3′ and 5′-CGACGCACTCTGTTGTCG-3.

### 4.6. Oxidative Stress Assays

Three replicates of the lipid peroxidation, catalase activity, and superoxide dismutase activity were measured by BIOXYTECH LPO-586 (OxisResearch, OXIS International, Beverly Hills, CA, USA), the Catalase Assay Kit (Cayman Chemical, Ann Arbor, MI, USA), and the Superoxide Dismutase Assay Kit (Cayman), respectively, following the manufacturers instructions. Briefly, SOD activity was assessed by measuring the dismutation of superoxide radicals generated by kit-supplied xanthine oxidase, and was detected by transforming tetrazolium salt into formazan dye, then quantified by absorbance at 440–460 nm. CAT activity was assessed by measuring the formaldehyde formation from methanol in the presence of H_2_O_2_ catalyzed by CAT, detected by the kit-supplied chromogenic 4-amino-3-hydrazino-5-mercapto-1,2,4-triazole, then quantified by the absorbance at 540 nm. Lipid peroxidation activity was assessed by measuring the decomposition of polyunsaturated fatty acid peroxides into malonaldehyde (MDA) and 4-hydroxyalkenals, detected by the kit-supplied chromogenic N-methyl-2-phenylindole, then quantified by absorbance at 586 nm. For the SOD, Cat, and LPO assays, the number of whole homogenized flies of each replicate was 50, 50, and 100, respectively.

### 4.7. Statistic Analysis

The significance of the difference between the survival curves and climbing curves of ergosta-7,9(11),22-trien-3β-ol treated and control groups were judged by log-rank (Mantel–Cox) and Gehan–Breslow–Wilcoxon tests. The significance of the difference between the performance index of learning memory of ergosta-7,9(11),22-trien-3β-ol treated and control groups were judged by Student’s *t*-test.

## 5. Conclusions

We verified that ergosta-7,9(11),22-trien-3β-ol (EK100) from Antrodia camphorata improves survival, learning, and memory in a Drosophila model of AD, with reduced biomarkers of microglia activation and inflammation. Moreover, the symptom modification did not occur through ubiquitous oxidative stress reduction, which means that ergosta-7,9(11),22-trien-3β-ol improves AD symptoms through different pathways to another Antrodia camphorate extract, antroquinonol. EK100 effectively decreased microglial activation and inflammation, which may help to reverse typical AD-associated cognitive deficits, such as learning and memory deficits.

## Data Availability

The data used to support the findings of this study are included in the article.

## Supplementary Materials

The following are available online. Figure S1: Survival of wild type flies treated with EK100; Figure S2: Climbing of wild type flies treated with EK100; Figure S3: Aversive odor avoidance of AD flies treated with EK100; Table S1: Statistics of the survival curves.

## Sample Availability

Samples of the compounds are available from the authors.

## References

[1] Davidson J.W. (1903). The Island of Formosa, Past and Present: History, People, Resources, and Commercial Prospects. Tea, Camphor, Sugar, Gold, Coal, Sulphur, Economical Plants, and Other Productions.

[2] Tsai Z., Liaw S. (1985). The Use and the Effect of Ganoderma.

[3] Chen C.-J. (2001). Study on solid cultivation and bioactivity of Antrodia camphorata. Fung. Sci..

[4] Geethangili M., Tzeng Y.-M. (2011). Review of pharmacological effects of *Antrodia camphorat*a and its bioactive compounds. Evid.-Based Complement. Altern. Med..

[5] Ogura S., Jakovljevic M.M. (2018). Global population aging-health care, social and economic consequences. Front. Public Health.

[6] Cummings J., Lee G., Ritter A., Zhong K. (2018). Alzheimer’s disease drug development pipeline: 2018. Alzheimer’s Dement. Transl. Res. Clin. Interv..

[7] Sharma P., Srivastava P., Seth A., Tripathi P.N., Banerjee A.G., Shrivastava S.K. (2019). Comprehensive review of mechanisms of pathogenesis involved in Alzheimer’s disease and potential therapeutic strategies. Prog. Neurobiol..

[8] Sochocka M., Donskow-Łysoniewska K., Diniz B.S., Kurpas D., Brzozowska E., Leszek J. (2019). The gut microbiome alterations and inflammation-driven pathogenesis of Alzheimer’s disease—A critical review. Mol. Neurobiol..

[9] Agostinho P., A Cunha R., Oliveira C. (2010). Neuroinflammation, oxidative stress and the pathogenesis of Alzheimer’s disease. Curr. Pharm. Des..

[10] Galasko D., Montine T.J. (2010). Biomarkers of oxidative damage and inflammation in Alzheimer’s disease. Biomark. Med..

[11] Kuo Y.-H., Lin T.-Y., You Y.-J., Wen K.-C., Sung P.-J., Chiang H.-M. (2016). Antiinflammatory and antiphotodamaging effects of ergostatrien-3β-ol, isolated from *Antrodia camphorata*, on hairless mouse skin. Molecules.

[12] Chao T.-Y., Hsieh C.-C., Hsu S.-M., Wan C.-H., Lian G.-T., Tseng Y.-H., Kuo Y.-H., Hsieh S.-C. (2021). Ergostatrien-3β-ol (EK100) from *Antrodia camphorata* Attenuates Oxidative Stress, Inflammation, and Liver Injury In Vitro and In Vivo. Prev. Nutr. Food Sci..

[13] Tsai T.-C., Tung Y.-T., Kuo Y.-H., Liao J.-W., Tsai H.-C., Chong K.-Y., Chen H.-L., Chen C.-M. (2015). Anti-Inflammatory Effects of Antrodia camphorata and its Active Compound, Ergostatrien-3β-ol, in a Mouse Skin Ischemia Model. FASEB J..

[14] Chang W.-H., Chen M.C., Cheng I.H. (2015). Antroquinonol lowers brain amyloid-β levels and improves spatial learning and memory in a transgenic mouse model of Alzheimer’s disease. Sci. Rep..

[15] Chen Y.-M., Sung H.-C., Kuo Y.-H., Hsu Y.-J., Huang C.-C., Liang H.-L. (2019). The Effects of Ergosta-7, 9 (11), 22-trien-3β-ol from *Antrodia camphorata* on the Biochemical Profile and Exercise Performance of Mice. Molecules.

[16] Finelli A., Kelkar A., Song H.-J., Yang H., Konsolaki M. (2004). A model for studying Alzheimer’s Aβ42-induced toxicity in *Drosophila melanogaster*. Mol. Cell. Neurosci..

[17] Ping Y., Hahm E.-T., Waro G., Song Q., Vo-Ba D.-A., Licursi A., Bao H., Ganoe L., Finch K., Tsunoda S. (2015). Linking aβ42-induced hyperexcitability to neurodegeneration, learning and motor deficits, and a shorter lifespan in an Alzheimer’s model. PLoS Genet..

[18] Rival T., Page R.M., Chandraratna D.S., Sendall T.J., Ryder E., Liu B., Lewis H., Rosahl T., Hider R., Camargo L. (2009). Fenton chemistry and oxidative stress mediate the toxicity of the β-amyloid peptide in a *Drosophila* model of Alzheimer’s disease. Eur. J. Neurosci..

[19] Ray A., Speese S.D., Logan M.A. (2017). Glial draper rescues Aβ toxicity in a *Drosophila* model of Alzheimer’s disease. J. Neurosci..

[20] Jeibmann A., Paulus W. (2009). *Drosophila melanogaster* as a model organism of brain diseases. Int. J. Mol. Sci..

[21] Brookmeyer R., Corrada M.M., Curriero F.C., Kawas C. (2002). Survival following a diagnosis of Alzheimer disease. Arch. Neurol..

[22] Lam L.C., Tang W., Leung V., Chiu H.F. (2001). Behavioral profile of Alzheimer’s disease in Chinese elderly–a validation study of the Chinese version of the Alzheimer’s disease behavioral pathology rating scale. Int. J. Geriatr. Psych..

[23] Schwab C., McGeer P.L. (2008). Inflammatory aspects of Alzheimer disease and other neurodegenerative disorders. J. Alzheimer’s Dis..

[24] MacDonald J.M., Beach M.G., Porpiglia E., Sheehan A.E., Watts R.J., Freeman M.R. (2006). The Drosophila cell corpse engulfment receptor Draper mediates glial clearance of severed axons. Neuron.

[25] Chen Z., Zhong C. (2014). Oxidative stress in Alzheimer’s disease. Neurosci. Bull..

[26] Stankovic N.D., Teodorczyk M., Ploen R., Zipp F., Schmidt M.H. (2016). Microglia–blood vessel interactions: A double-edged sword in brain pathologies. Acta Neuropathol..

[27] Konishi H., Kiyama H. (2018). Microglial TREM2/DAP12 signaling: A double-edged sword in neural diseases. Front. Cell. Neurosci..

[28] Sevigny J., Chiao P., Bussière T., Weinreb P.H., Williams L., Maier M., Dunstan R., Salloway S., Chen T., Ling Y. (2016). The antibody aducanumab reduces Aβ plaques in Alzheimer’s disease. Nature.

[29] Lalli G., Schott J.M., Hardy J., De Strooper B. (2021). Aducanumab: A new phase in therapeutic development for Alzheimer’s disease?. EMBO Mol. Med..

[30] Takata K., Kitamura Y., Saeki M., Terada M., Kagitani S., Kitamura R., Fujikawa Y., Maelicke A., Tomimoto H., Taniguchi T. (2010). Galantamine-induced amyloid-β clearance mediated via stimulation of microglial nicotinic acetylcholine receptors. J. Biol. Chem..

[31] Mandrekar-Colucci S., Karlo J.C., Landreth G.E. (2012). Mechanisms underlying the rapid peroxisome proliferator-activated receptor-γ-mediated amyloid clearance and reversal of cognitive deficits in a murine model of Alzheimer’s disease. J. Neurosci..

[32] McGeer P.L., McGeer E.G. (2015). Targeting microglia for the treatment of Alzheimer’s disease. Expert Opin. Ther. Targets.

[33] Song W.M., Colonna M. (2018). The identity and function of microglia in neurodegeneration. Nat. Immunol..

[34] Kuo Y.-H., Lin C.-H., Shih C.-C. (2015). Ergostatrien-3β-ol from *Antrodia camphorata* inhibits diabetes and hyperlipidemia in high-fat-diet treated mice via regulation of hepatic related genes, glucose transporter 4, and AMP-activated protein kinase phosphorylation. J. Agric. Food Chem..

[35] Le Bourg E., Lints F.A. (1992). Hypergravity and aging in Drosophila melanogaster. 4. Climbing activity. Gerontology.

[36] Waddell S., Quinn W.G. (2001). Flies, genes, and learning. Ann. Rev. Neurosci..

