# Supplementary material for: Ergosta-7,9(11),22-trien-3β-ol Rescues AD Deficits by Modulating Microglia Activation but Not Oxidative Stress
Source: Molecules. 2021 Sep 2;26(17):5338. doi: 10.3390/molecules26175338 (PMC8433642; doi:10.3390/molecules26175338)
Supplement: Supplementary file 1 [file molecules-26-05338-s001.zip › molecules-1325367-supplementary.pptx]

## Slide 1
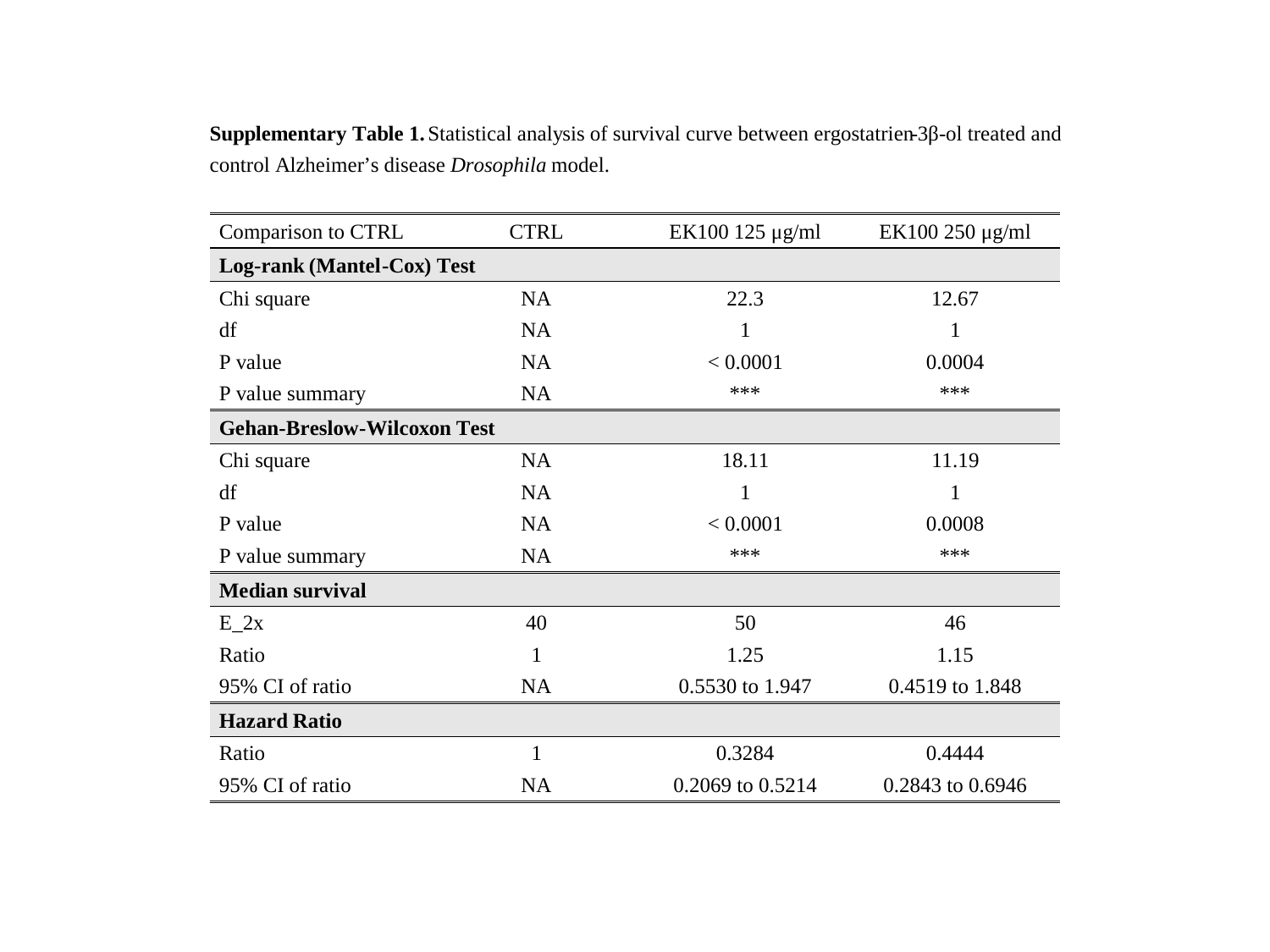

## Slide 2
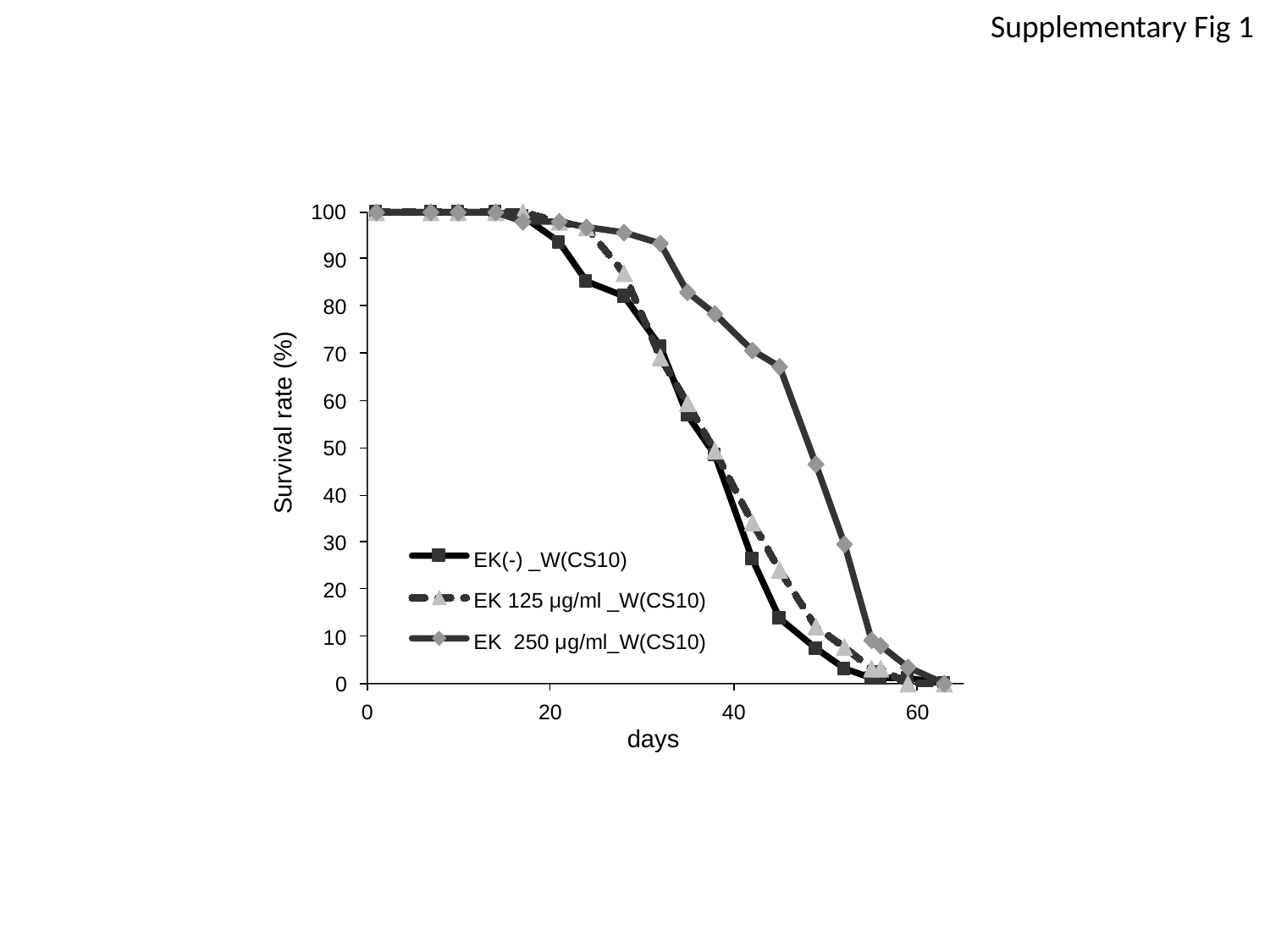

Supplementary Fig 1
100
90
80
70
60
50
40
30
20
10
0
0
20
40
60
Survival rate (%)
EK(-) _W(CS10)
EK 125 μg/ml _W(CS10)
EK 250 μg/ml_W(CS10)
days

## Slide 3
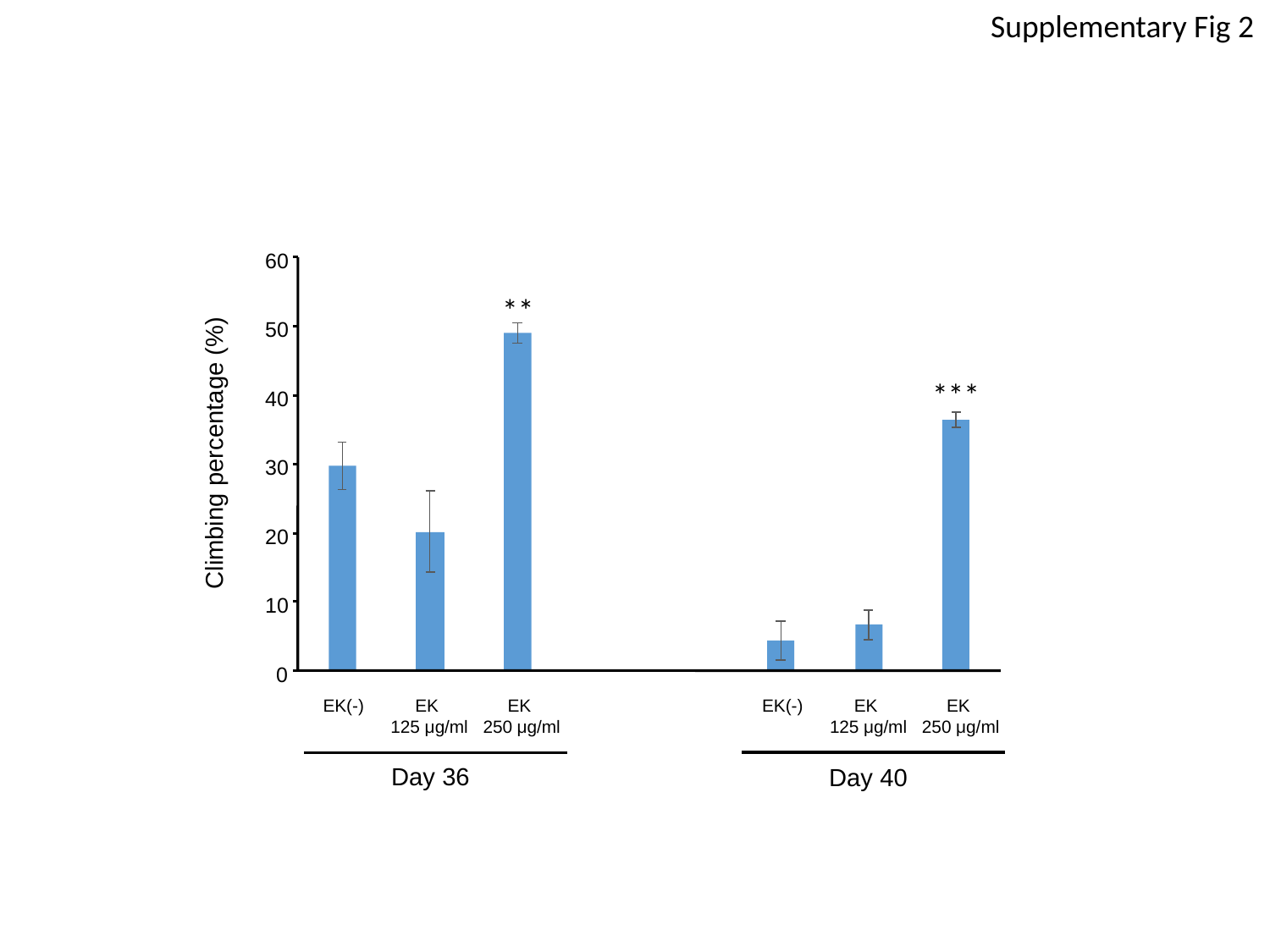

Supplementary Fig 2
60
**
50
***
40
Climbing percentage (%)
30
20
10
0
EK(-)
EK
125 μg/ml
EK
250 μg/ml
EK(-)
EK
125 μg/ml
EK
250 μg/ml
Day 36
Day 40

## Slide 4
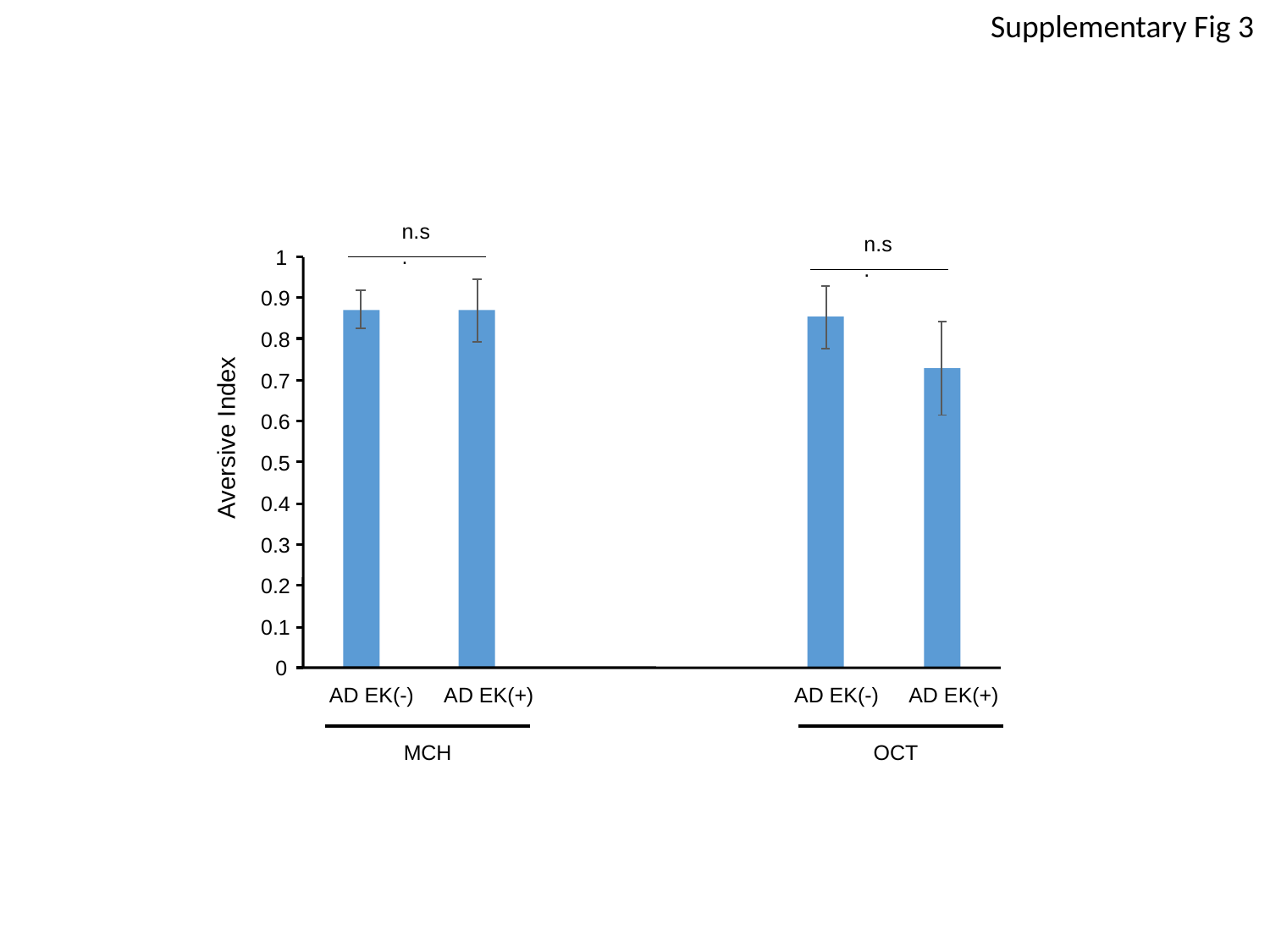

Supplementary Fig 3
n.s.
n.s.
1
0.9
0.8
0.7
Aversive Index
0.6
0.5
0.4
0.3
0.2
0.1
0
AD EK(-)
AD EK(+)
AD EK(-)
AD EK(+)
MCH
OCT
